# Behavioral adjustments and support use of François' langur in limestone habitat in Fusui, China: Implications for behavioral thermoregulation

**DOI:** 10.1002/ece3.6249

**Published:** 2020-04-15

**Authors:** Youbang Li, Xiaohong Huang, Zhonghao Huang

**Affiliations:** ^1^ Key Laboratory of Ecology of Rare and Endangered Species and Environmental Protection (Guangxi Normal University) Ministry of Education Guilin China; ^2^ Guangxi Key Laboratory of Rare and Endangered Animal Ecology Guangxi Normal University Guilin China

**Keywords:** behavioral adaptation, support use, thermal stress, thermoregulation, *Trachypithecus francoisi*

## Abstract

Climatic factors such as temperature and humidity vary seasonally in primate habitats; thus, behavioral adjustments and microhabitat selection by primate species have been interpreted as behavioral adaptations. François' langur (*Trachypithecus francoisi*), a native species to southwest China and northern Vietnam, inhabits a limestone habitat with extreme climatic conditions. To understand the potential effects of climatic seasonality on this species, we collected data on the individual behavioral budgets in a *T. francoisi* group between January and December 2010 in Fusui County, China. Monthly, we performed 5–11 days of observation during this period, using focal animal sampling and continuous recording methods. We also recorded ambient temperature (*T*_a_) and relative humidity (*H*_r_) data at our study site. Results indicated that *T*_a_ and *H*_r_ were significantly correlated with each other and fluctuated dramatically on a daily, monthly, and seasonal basis. The amount of time spent resting, grooming, basking, and huddling also varied on a daily, monthly, and seasonal basis. The proportion of resting time and total sedentary activity time significantly increased at high and low *T*_a_s, respectively. The total sedentary time, resting time, and plant branch use all showed positive significant correlations with *T*_a_. Our results suggest that behavioral adjustment and support use of *T. francoisi*, at least partly, were related to thermoregulation. *T. francoisi* minimized thermal stress through behavioral adjustments and support use. It is an adaptive behavior associated with the climatic extremes of limestone habitat. This study can potentially advise conservation management strategies in this specific habitat. Conservation efforts should focus on vegetation restoration in langurs' habitat, including those in the foothills.

## Graphical abstract

The habitat of the François' langur in this study was characterized by dramatic climatic ranges. François' langur minimized thermal stress and increased physical comfort by either behavioral adjustment or alternation of support use.

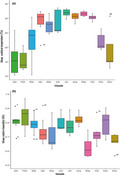

## 1 INTRODUCTION

Most physiological processes are sensitive to environmental changes and perform optimally within a narrow range of conditions. Small changes in environmental factors (e.g., temperature) can potentially impact the efficiency of biological systems (Whiteman & Buschhaus, 2003). In turn, environmental factors are known to alter animal behavior. In most animals, high ambient temperatures (*T*_a_) cause thermal stress, and humidity further exacerbates this effect (Blackshaw & Blackshaw, 1994). Few organisms can maintain a sufficiently high metabolic rate at very low or very high temperatures (Stiling, 1996), and such extreme temperatures can thus be lethal (Smith, 1958). However, most animals can actively acclimate to environmental changes via physiological and/or behavioral means (Ma, Bai, Wang, Majeed, & Ma, 2018). A substantial amount of research on the influence of climatic factors (temperature, humidity, sun altitude, shade) on primate behavioral adjustments and habitat selection has been conducted by primatologists (Clutton‐Brock, 1975; Fan, Ni, Sun, Huang, & Jiang, 2008; Hafez, 1964; Hill, Weingrill, & Barrett, 2004; Huang, Wei, Li, Li, & Sun, 2003; Majolo, McFarland, Young, & Qarro, 2013; Sato, 2012; Stelzner, 1988; Stelzner & Hausfater, 1986). The seasonal variation in activities and microhabitat use is thought to be related to thermoregulation in a number of primates (e.g., *Alouatta palliate*: Thompson, Williams, Glander, & Vinyard, 2016). For instance, when *T*_a_ is high, primates reduce their daily travel distance (McLester, Brown, Stewart, & Piel, 2019), spend more time resting in the shade (*Callithrix jacchus*: Abreu, De la Fuente, Schiel, & Souto, 2016; Fuente, Souto, Sampaio, & Schiel, 2014; *Colobus polykomos*: Dasilva, 1992; *Papio cynocephalus:* Stelzner, 1988), and stay in cooler microhabitats (e.g., caves) during the day (*Pan troglodytes verus*: Pruetz, 2007; *A. palliate*: Thompson et al., 2016). On the other hand, when *T*_a_ is low, primates respond by adopting alternative behaviors: They may change social relationships (increasing physical contact with others) within their group to benefit their thermal competences (McFarland et al., 2015), adjust nest architecture to increase thermoregulation (Stewart, Piel, Azkarate, & Pruetz, 2018), preferentially use heat‐conserving postures in sunny areas or stay under direct sunlight (*Alouatta caraya:* Bicca‐Marques & Calegaro‐Marques, 1998; *Callicebus nigrifrons:* Gestich, Caselli, & Setz, 2014; *P. cynocephalus:* Stelzner & Hausfater, 1986), form huddles (*Macaca fuscata:* Ogawa & Wada, 2011; Ueno & Nakamichi, 2018), select warmer microhabitats during the day (*A. palliate:* Thompson et al., 2016), or remain for longer in caves (*P. hamadryas ursinus:* Barrett, Gaynor, Rendall, Mitchell, & Henzi, 2004). As summarized by Dunbar, Korstjens, and Lehmann (2009), primates actively avoid thermal stress (e.g., avoid being exposed to environments with *T*_a_s that are too high or too low) through various behavioral adjustments. These adjustments enable individuals to acclimate to extreme climatic variability while minimizing their metabolic costs (Aujard et al., 2006; Gordon, Fehlner, & Long, 1986). Resting time provides the energetic “capital” from which time budget allocation is made. According to Dunbar et al. (2009), the amount of resting time required is determined by two primary sources of energy expenditures: digestion and *T*_a_. In ruminants, animals minimize physical activity during digestion, due to the heat load generated by fermentation and because bacteria responsible for fermentation in ruminant folivores are temperature‐sensitive (van Soest, 1982). When *T*_a_ rises significantly above a species' thermoneutral zone, animals are forced to remain in the shade to avoid thermal overload; conversely, when *T*_a_ drops significantly below a species' thermoneutral zone, the animal needs to spend additional time resting to conserve heat and offset the costs of thermoregulation (Mount, 1979). Thus, the study of temporal variation in time budgets in relation to contextual environmental factors can further our understanding of how environmental factors affect animal behavior and the strategies that animals use in response to this variation.

François' langur (*Trachypithecus francoisi*) is an endangered primate species (IUCN, 2008) endemic to the limestone forests of southwest China and northern Vietnam (Li, Huang, Ding, Tang, & Wood, 2007; Nadler, Momberg, Dang, & Lormee, 2003). Limestone habitat has some distinctive natural features. Firstly, a limestone habitat is characterized by extreme diurnal fluctuations in temperature in all seasons (up to 30°C in range) (Huang, Li, Zhou, & Wei, 2003). During a sunny day, *T*_a_ increases rapidly after sunrise and peaks around 14:00; then, *T*_a_ decreases gradually until about 4:00 in the morning (C. M. Huang, unpublished). Primates living in habitats with such strong temperature fluctuations can be energetically challenged (Kobbe, Nowack, & Dausmann, 2014): They are likely to be highly susceptible to thermoregulatory costs (Hill, 2006). Secondly, limestone forests tend to be dominated by drought‐enduring plants (Liu, Groves, Yu, & Meiman, 2004), which have been selectively harvested by local populations, resulting in more open, fragmented environments with short vegetation (Huang, Wei, Zhou, Li, & Huang, 2007). Habitat fragmentation is known to alter thermal conditions, which strongly influence species' activity patterns (Tuff, Tuff, & Davies, 2016). Therefore, animals inhibiting limestone habitats are expected to adopt various strategies to cope with such thermal conditions, including behavioral adjustments. Lastly, limestone habitats are highly heterogeneous, containing several support types such as plant branches, bare rocks, and caves (Huang, Li, Zhou, & Wei, 2004), which in turn correspond to high spatial heterogeneity in thermal conditions, providing a range of microhabitats for animals to exploit (Thompson et al., 2016). For example, the shade created by tree branches is an important microhabitat for animals seeking to avoid direct sunlight during warm days (Abreu et al., 2016). In this study, we conducted research focusing on climatic factors, behavioral patterns, and support use in a group of *T. francoisi* living in a limestone habitat to test the following hypotheses:

1. As mentioned before, most primate species in habitats with large temperature extremes use behavioral adjustment (e.g., resting, basking) in response to thermal stress. Folivorous *T. francoisi* langurs are very susceptible to runny noses, coughing, sneezing, and other respiratory diseases caused by dramatic temperature changes within their habitat (Tian & Zhang, 1993). Therefore, we hypothesized that they might have some behavioral adjustments (e.g., resting, basking, huddling) to reduce this energy cost in limestone habitats characterized by large fluctuations in temperature.
2. For limestone langurs (*Trachypithecus* genus), substrate use is similar. Some studies imply that several substrates (e.g., cave and bare rock) are used more frequently during cold and rainy days (Huang et al., 2004; Workman, 2010). Therefore, we hypothesize that the proportion of support use (e.g., branch, cave, bare rock) of langurs is related to thermoregulation.

## 2 MATERIALS AND METHODS

### 2.1 Study site and objectives

Our chosen study site is a 25.7‐ha limestone hill fragment (107°50′ E, 22°45′ N) in Fusui County, China. This area is surrounded by farmland, and observers could easily walk around the hillsides. The site is characterized by a limestone landscape, rich in steep cliffs, natural caves, and overhangs. The site is situated within the subtropical monsoon climate zone, with four seasons: spring (March–May), summer (June–August), autumn (September–November), and winter (December–February) (Fusui County Annals Committee, 1989). The average daily temperature was 22.1°C (range: −0.5–39.5°C), and the average annual precipitation was 1,054.3 mm (range: 1,022–1,769 mm). A previous study showed that the site has two distinct seasons: a rainy season ranging from April to September with ≥50 mm of monthly rainfall and a dry season in the remainder of the year, with <50 mm of monthly rainfall (Zhou, Huang, Wei, Wei, & Huang, 2009). This area receives 81.4% of its annual rainfall during the rainy season when the mean monthly precipitation is 171.7 mm (range: 123.7–228.7 mm). A continuous and selective firewood harvest by local populations has removed the larger trees, thereby significantly altering the vegetation composition and creating more open areas (dominated by bare rocks); thus, the vegetation is now characterized as a secondary broadleaf forest (Xue, 2000). Small remnants of forest remained along the steep slopes of the hillsides, where no harvesting is possible. Langurs were observed to predominantly use five caves as night sleeping sites.

The study focused on a relatively well‐habituated group of François's langurs. The group was made up of four adults (one male and three females). Individuals were easily identifiable for most of the time on the basis of sex, body size, and tail length. The minimum distance the group tolerated to the presence of observers was 30 m. The average distance between the group and the observers throughout this study was about 100 m.

### 2.2 Field observation

We observed the behavioral patterns and the related supports of individual langurs during the first 12 days of each month between January and December 2010. For each observation day, observation sessions began when animals left their sleeping sites and ended when animals entered their sleeping sites. We divided the observation time into 15‐min intervals. Using focal animal sampling and continuous recording (Altmann, 1974; Martin & Bateson, 1986), we focused on one individual in the first 5 min of each 15‐min unit and continuously recorded the time the animal spent performing each behavior and which support type was used. We avoided repeatedly sampling conspicuous animals by moving through the group when selecting subjects and by sampling both animals that were in clear view and those that were more hidden. This strategy ensured that each sample was discreet form the previous one, even if the same individual was sometimes sampled again in the subsequent time unit (Stanford, 1991). We conducted field observation using binoculars (Panda 10 × 40) and a spotting scope (Nikon Fieldscope ED82, 25‐75X Zoom) at distances ranging between 30 and 200 m.

Studies have shown that more sedentary activities are related to thermal stress (Hill et al., 2004; Pochron, 2000; Stelzner, 1988), while feeding and traveling are generally assumed to be heat‐generating activities (Dunbar et al., 2009). In this study, we observed the following behavioral patterns: grooming (both self‐grooming and allogrooming), resting, huddling and basking, and total sedentary activity budget (the sum of resting, grooming, huddling, and basking). The position of the animals did not change when these behaviors occurred. Support types used during these behaviors (e.g., plant branch, cave, bare rock, and grass/soil) were identified. The behavioral pattern and support use categories used in this study are shown in Table 1. As pointed out by Sade (1972), activities tend to be polythetic, meaning that one behavior may be included in several behavior categories. For example, when animals are grooming (both self‐grooming and allogrooming) in the sun in the morning, the behaviors can be assigned to basking and grooming, as well as resting. In such cases, we followed the methods of Sade (1972) and Dunbar (1992) by hierarchically assigning the behavior to specific behavior categories. Huang, Wei, et al. (2003) argue that basking is a form of resting and that it should be recorded separately, because it is an important behavior in langurs living in limestone. Thus, basking was chosen when animals were inactive and in the sunshine, and it was assumed to take precedence over all other activities. The second behavior was huddling, easily distinguished as physical contact between individuals. From observations in other primates, huddling is thought to have thermoregulatory properties (e.g., *M. fuscata:* Ueno & Nakamichi, 2018). The third and fourth behaviors in this hierarchy were grooming and resting, respectively. Like other limestone langurs (Hendershott, Rawson, & Behie, 2018; Liu et al., 2004; Workman, 2010), langurs were hidden around mid‐day, though their locations were confirmed. The observations were not conducted during this time in some studies (Hendershott et al., 2018; Liu et al., 2004). While other studies conducted the behavioral observation when the location of langurs could be confirmed and inferred that animals were considered to be resting when they hid in vegetation (Huang, Wei, et al., 2003; Zhou, Huang, & Fan, 2001), the vegetation in these types of habitat tends to be dominated by shrubs and tree saplings, with branches generally not being able to support several animals grooming each other, or shake strongly. Following this method, when individuals were hidden from the observer by trees, shrubs, or caves, but the location of the focal animals could be confirmed, these records were assigned as resting. This can mean, however, that our data are likely biased toward rest. Similarly, support use was assigned in a hierarchy. If animals used caves as support during the day, then priority would be given to “cave” whether sitting or lying on rocks or resting in rifts. The second and third support use was bare rock and grass/soil. The last support category was plant branch. However, owing to the special topographic feature of limestone habitat, it is possible for a langur to be on rock under dense tree crown (Hendershott et al., 2018). Thus, if langurs used rocks or grass/soil covered by vegetation or ledge, we recoded it as “shade.” Based on the fact that primates seek shade as an important microhabitat to avoid direct sunlight during warm days (Abreu et al., 2016; Duncan & Pillay, 2013), we lumped “shade” and branch use together for further analysis.

**TABLE 1:** Description of behavioral patterns and support types

Items	Description	References
I Behavioral patterns		
Resting	Whether sitting‐and‐looking, standing, sitting, or lying on supports, the individual did not move for at least 30 s; or whenever the individual was hidden from the observer by trees, shrubs, or caves, but its location could be confirmed	Dunbar (1992); Huang, Wei, et al. (2003); Struhsaker (1975); Vogt (1978)
Self‐grooming	When the focal individual examines its fur and removes particles or parasites by itself and its position remains unchanged	Hill et al. (2004); Struhsaker (1975)
Allogrooming	When one individual groomed another (and the focal individual was involved)	Vogt (1978)
Huddling	When two or more individuals were resting in physical contact with one another	Ogawa and Wada (2011)
Basking	Sitting‐and‐looking, standing, sitting or lying, on rocks, or soil/grass, as well as above the canopy where the sunshine could reach directly the individual. Under such circumstances, individuals usually changed postures and orientations to maximize sun exposure	Stelzner and Hausfater (1986)
II Support types		
Plant branch	Mainly in trees and shrubs, where the sunshine did not reach the individual when stationary	
Cave	Animals were in caves during the daytime, even if the animals were sitting on rocks or resting in rifts while inside the cave	
Bare rock	Rock on which the animals were sitting, lying, or grooming. Specially, rock on which langur sit was shaded by trees or ledge, the support category was recorded as “shade.”	
Grass/soil	Animals on grass/soil during the daytime. Specially, grass/soil on which langur sit was shaded by trees or ledge, the support category was recorded as “shade.”	

Throughout the observation period, langurs on 53 days (monthly ranging from 1 to 7 days) were lost or only followed for half a day, due to the harsh terrain. Langur group was fully followed (from the beginning when animals left their sleeping sites and the ending when animals entered their sleeping sites) on 91 days (monthly observation days ranged from 5 to 11 days), with a total observation time of 1,045.5 hr (Table 2). 

**TABLE 2:** Monthly behavioral time budget and support use of *Trachypithecus francoisi* during the study period (January–December 2010)

Season	Month	Observation days	Behavioral time budget (%)					Support use (%)		
			Resting	Grooming	Basking	Huddling	Total sedentary	Plant branch	Cave	Bare rock
Winter	January	9	53.7 (22.1)	5.1 (4.3)	6.9 (20.8)	1.6 (4.7)	67.3 (14.11)	48.3 (24.5)	6.9 (13.8)	12.1(22.2)
	February	11	60.0 (18.1)	1.7 (2.0)	0.8 (2.6)	0.0	62.5 (10.2)	38.9 (20.9)	19.1 (21.0)	4.5 (3.7)
Spring	March	11	54.2 (9.4)	4.0 (3.0)	0.0	0.0	58.2 (7.3)	52.3 (6.8)	1.8 (6.1)	4.1 (3.0)
	April	10	62.6 (10.4)	2.3 (2.0)	0.0	0.0	64.9 (9.9)	47.5 (21.8)	15.1 (20.2)	2.3 (2.0)
	May	7	63.0 (7.9)	1.3 (2.0)	0.0	0.0	64.3 (8.1)	63.0 (16.0)	0.0	1.3 (1.6)
Summer	June	7	70.5 (8.3)	2.3 (1.5)	0.0	0.0	72.8 (7.7)	70.5 (14.8)	0.0	2.3 (1.4)
	July	6	64.5 (6.8)	1.7 (1.6)	0.0	0.0	66.2 (6.1)	64.5 (16.4)	0.0	1.7 (1.6)
	August	8	64.3 (7.9)	1.0 (1.1)	0.0	0.0	65.3 (8.6)	64.3 (11.2)	0.0	1.0 (1.1)
Autumn	September	6	75.2 (5.6)	1.1 (0.9)	0.0	0.0	76.3 (5.6)	53.1 (22.3)	22.3 (22.7)	1.1 (0.8)
	October	6	79.8 (7.0)	0.7 (1.3)	0.0	0.0	80.5 (6.0)	57.7 (25.2)	22.2 (24.4)	0.7 (1.2)
	November	5	33.2 (13.7)	12.3 (7.1)	11.1 (15.4)	0.0	56.6 (5.4)	28.0 (11.0)	5.2 (11.7)	23.4 (9.7)
Winter	December	5	26.4 (14.6)	13.6 (4.8)	9.4 (6.3)	9.5 (21.3)	58.9 (9.1)	35.9 (4.4)	0.0	23.0 (4.9)
	Total	91	59.7 (17.4)	3.9 (4.5)	1.9 (8.0)	0.7 (5.2)	66.2 (11.0)	52.0 (17.8)	7.7 (20.9)	6.5 (10.0)

Note

*SD* is shown in parentheses.

### 2.3 Measurement of climatic variables

Ambient temperature (*T*_a_) and relative humidity (*H*_r_) were recorded during each 15‐min behavioral observation, using a portable thermometer **(**RC‐T601A, Changzhou Nuke Instrument Co.LTD). In general, the focal group always moved within its home range; accordingly, observers needed to find several optimal observation sites and relocated the thermometer with each new daily movement of the group. As the isolated hill was small, we assumed that there was no evident variation in conditions around the hill. As pointed out by Stelzner and Hausfater (1986), air temperature is of the greatest microclimatic importance for individuals in trees. It thus seems safe to assume that meteorological measurements taken above ground‐level were strongly correlated with the conditions actually experienced by the langurs. Previous observations have shown that langurs mainly confined their locomotion to the main canopy, except when they were resting on rocks (Zhou, Luo, Wei, & Huang, 2013). Therefore, we adopted Stelzner and Hausfater's (1986) design to measure temperature and relative humidity 30 cm above the ground. We avoided exposing the thermometer to direct sunshine and recorded weather variables for every observation day. Weather conditions were roughly classified as sunny, rainy, cloudy, cloudy/sunny, or cloudy/rainy. The observation days included 45 sunny days (47.4% of the total observation days), 12 cloudy/sunny days (12.6%), 20 cloudy days (21.1%), nine rainy days, and nine cloudy/rainy days (9.5%).

### 2.4 Data analysis

We calculated mean daily temperature (*T*_a_) and relative humidity (*H*_r_) by averaging daily data. In the same way, we also calculated mean seasonal *T*_a_ and *H*_r_.

We tried to avoid repeatedly sampling conspicuous animals by moving through the group when selecting new subjects and by sampling both animals that were in clear view and those that were hidden. However, for much of the observation duration, individual identification was hard due to the harsh terrain (observers were unable to reach some sites where langurs were) and the morphological similarity between individuals, as well as the distance between the animals and observers. We were not able to calculate proportions separately for each focal animal; thus, we followed the method used by Huang, Wei, et al. (2003), Huang, Wu, Zhou, Li, and Cai (2008) and lumped all the observations together. In this study, only data collected from full observation days were included in the analysis. We used descriptive statistics to examine proportions of observations of behavioral time budget and the support use of individuals. Following Struhsaker (1975) who recorded groomer and groomee in the grooming category, we recorded self‐grooming and allogrooming within the grooming category. Additionally, resting, grooming, basking, and huddling were all characterized by a static stance throughout the behavior, although orientation may have changed; thus, the time devoted to these behaviors was summed up to represent total sedentary activity budget time.

Observation days varied among months, and diurnal sampling time varied across days. Following Post (1981), we calculated the diurnal behavioral time budget for each behavioral pattern (e.g., resting) for each day by dividing the behavioral time budget by the total sample duration of a day; then, we averaged the means across each observation day within a specific month to calculate monthly proportions, which were further averaged across months to calculate seasonal and total behavioral time proportions. Similarly, we calculated daily, monthly, seasonal, and annual proportions of time spent on each support type (branch, cave, bare rock). All the values were reported as mean ± *SD*.

Zuur, Leno, and Elphick (2010) argue that high, or even moderate, collinearity is especially problematic when ecological signals are weak. Collinearity can be expected if temporal variables (e.g., month, year) are used together with covariates. We used the Kendall rank test to detect collinearity between climatic variables and found that *T*_a_ and *H*_r_ were collinear (Kendall rank *τ* *=* −0.17, *p* = .03). Therefore, we dropped the collinear covariates before proceeding with further analysis. Research has shown that temperature represents an important ecological constraint on primates (Hill et al., 2004). Thus, we used *T*_a_ to analyze the effects of climatic variables on activity time budget using a generalized linear model (GLM), where *T*_a_ was set as independent variable, and resting, grooming, basking, and huddling behaviors were dependent variables. In the same way, we analyzed the effects of *T*_a_ on support use, but the use of grass/soil was excluded from the analysis, as the proportion of their support use was <1%. In order to meet the assumptions of statistical tests, we used an arcsine transformation on all proportions to stabilize the variance. The model with the minimum Akaike information criterion (AIC) was selected as the best‐fit model to assess the relative effects of different thermal variables (Thompson et al., 2016).

Additionally, following Campos and Fedigan (2009), we defined the *T*_a_ category boundary as +1 and −1 *SD* from the mean of all systematically collected daily mean *T*_a_. *T*_a_s was classed in the following categories limits: (a) low: *T*_a_ ≤ 17.314°C, (b) medium: 17.314°C < *T*_a_ < 32.172°C, and (c) high *T*_a_ ≥ 32.172°C. As the variance in proportion for some behaviors still failed to meet the normality assumptions required for parametric tests, Friedman's test was used to analyze whether there were significant variations in given behavioral budgets (total sedentary, resting, grooming, huddling, basking) and a given support use (branch, bare rock, cave) at the three *T*_a_s categories (low, medium, and high) and post hoc Wilcoxon tests were used to examine pairwise differences whenever Friedman's test was significant.

One‐sample *t* tests were used to analyze daily variation in *T*_a_, *H*_r_, and in the behavioral time budget (including total sedentary budget, resting, grooming, huddling, and basking), as well as time spent on various support types. We used one‐way ANOVA to examine whether there were monthly and seasonal variations in climatic variables, behavioral adjustments, and support use. We also calculated the Spearman correlation between total sedentary behaviors and *T*_a_. We conducted the tests in R for windows version 3.3.0 (The R Foundation for Statistical Computing, 2016). The significance level was set at *p* = .05.

## 3 RESULTS

### 3.1 Variations in *T*_a_ and *H*_r_

**FIGURE 1:**
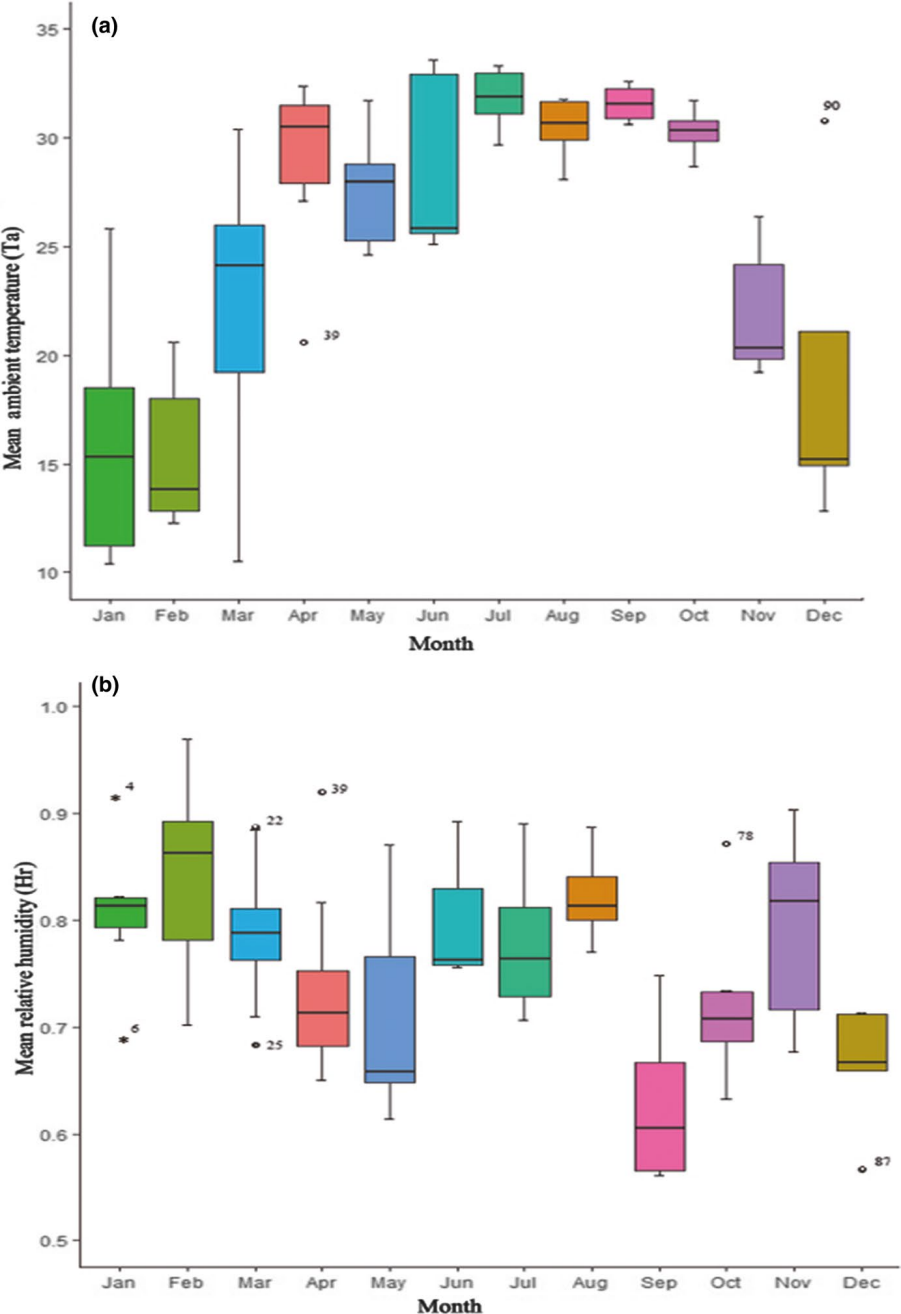
Monthly ambient temperature (°C) (a) and relative humidity (%) (b) of the study site between January and December 2010. Outliers are indicated by asterisk or cycles

On a daily basis, *T*_a_ was low in the morning and high at noon, while *H*_r_ showed an opposite trend. The lowest *T*_a_ recorded was at 7:00, when langurs came out from their night‐staying cave. *T*_a_ gradually increased and peaked between 12:00 and 17:00. In contrast, *H*_r_ was highest in the early morning and lowest between 12:00 and 17:00. On a monthly basis, *T*_a_ was high during the middle months (June–July) of the year and H_r_ fluctuated throughout the observation duration (Figure 1). On a seasonal basis, on average summer had the hottest T_a_ with a value of 30.9 ± 1.5°C and winter had the coldest *T*_a_ with an average of 14.6 ± 4.0°C. *H*_r_ peaked four times during the total observation study. The overall mean monthly *T*_a_ and *H*_r_ were 25.3 ± 7.4°C (ranging from 14.4°C in January to 33.6°C in July) and 76.7 ± 9.8% (ranging from 63.1 ± 5.6% in December to 86.7 ± 6.2% in June), respectively. Both *T*_a_ and *H*_r_ varied significantly between months (ANOVA: *T*_a_: *F* = 28.87, *df* = 11, *p* < .01; *H*_r_: *F* = 5.91, *df* = 11, *p* < .01) and seasons (ANOVA: *T*_a_: *F* = 30.65, *df* = 3, *p* < .001; *H*_r_: *F* = 7.73, *df* = 3, *p* < .001).

### 3.2 Behavioral time budget and temporal variations

Overall, the mean total sedentary budget for langurs was 66.2 ± 11.0%, ranging from 56.5 ± 5.4% in November to 80.5 ± 6.0% in October (Table 2). Total sedentary budget varied significantly on a daily (*t* = 55.90, *df* = 90, *p* < .01), monthly (ANOVA: *F* = 5.67, *df* = 11, *p* < .01), and seasonal basis (ANOVA: *F* = 5.91, *df* = 3, *p* = .001). The total sedentary budget was positively and significantly correlated with *T*_a_ (*r* = .35, *p* < .01, *N* = 91).

Langurs spent most of their time resting. The mean proportion of time spent resting was 59.7 ± 17.4%, ranging from 26.4 ± 14.6% in December to 79.8 ± 7.0% in October. Grooming occurred in all seasons but accounted for a smaller proportion of the activity budget. Basking and huddling occurred only in winter and late autumn (Table 2).

The proportion of time spent resting and total sedentary activity were both higher at low and high *T*_a_s, while grooming was more frequent at medium *T*_a_. Huddling occurred only at low *T*_a_. Basking occurred at both low and medium *T*_a_s (Table 3). Within the resting category, significant differences were found between low and medium, low and high *T*_a_s. Within the sedentary category, a significant difference was found between low and medium *T*_a_s. However, no significant difference in the proportion of time allocated to basking, huddling, or grooming was observed among the three temperature categories (Table 3).

**TABLE 3:** Friedman's tests of behavioral budget (%) differences and support use of *Trachypithecus francoisi* at low, medium, and high temperatures during the study period (between January and December 2010)

	Temperature category^a^			*N*	Chi‐square	*df*	*p*	Post hoc Wilcoxon tests *p*‐values		
	Low	Medium	High					Low–Med	Low–High	Med–High
Behavioral budget (%)										
Total sedentary	64.4 (12.0)	56.6 (6.7)	70.8 (7.1)	12	11.167	2	.004*	.044*	.07	.133
Resting	57.4 (12.6)	49.7 (13.1)	67.8 (7.5)	12	8.665	2	.013*	<.001*	<.001*	.163
Grooming	3.1 (3.8)	5.2 (5.3)	3.0 (2.4)	12	3.168	2	.205			
Huddling	3.7 (12.7)	0.0	0.0	12	2.000	2	.368			
Basking	1.6 (3.7)	1.6 (4.1)	0.0	12	2.000	2	.368			
Support use (%)										
Plant branch	44.0 (22.0)	42.7 (18.4)	51.8 (24.5)	12	2.000	2	.368			
Bare rock	39.4 (24.3)	53.4 (16.4)	39.0 (17.7)	12	1.167	2	.558			
Cave	16.6 (21.3)	3.9 (9.1)	9.3 (15.6)	12	1.613	2	.446			

Note

*SD* shown in parentheses.

^a^ Temperature categories were as follows: low: *T*_a_ ≤ 17.314°C; medium: 17.314°C < *T*_a_ < 32.172°C; high *T*_a_ ≥ 32.172°C.

* Differences are significant at .05 levels.

Based on AIC, the best‐fit model selected for *T*_a_ included only resting as a dependent variable (rest = 14.10 + 16.96 × *T*_a_, AIC = 339.23, *df* = 86, *F* = 6.14, *p* = .002), but not grooming, basking, and huddling, suggesting that resting was significantly affected by *T*_a_, while other behaviors were not.

### 3.3 Variation in support use

Langurs spent 52.0 ± 17.8% (range: 28.0%–64.5%) of the observation time on branches (Table 2). There were significant differences in time spent in branches on a daily (*t* = 24.25, *df* = 90, *p* < .01), monthly (ANOVA: *F* = 3.58, *df* = 11, *p* < .01), and seasonal basis (ANOVA: *F* = 9.39, *df* = 3, *p* < .01). Langurs used branches less often at medium *T*_a_ (Table 3).

Only two caves in steep cliffs were used during the daytime, but were not known to have been used at night. Cave use was not evenly spread across months. Cave use peaked in winter and in warm autumn days (Table 2). Cave use in winter mainly occurred on rainy or cloud/rain days, while cave use in autumn exclusively occurred on sunny days. Monthly cave use ranged from 0 to 22.3 ± 22.7%, and the mean proportion of time using the cave peaked twice, once at high *T*_a_ (>30°C) and once at low Ta (10–15°C), accounting for 32.2 ± 20.4% and 42.9 ± 19.5% of total cave use, respectively. There was significant variation in daily (*t* = 4.84, *df* = 90, *p* < .01), monthly (ANOVA: *F* = 3.18, *df* = 11, *p* = .001), and seasonal cave use (ANOVA: *F* = 4.53, *df* = 3, *p* = .005). Langurs spent longer in their caves at low *T*_a_ (Table 3).

Monthly bare rock use ranged from 0.7 ± 1.2% to 23.4 ± 9.7% (Table 2). Bare rock usage most frequently occurred when *T*_a_ was 10–20°C and rarely occurred when *T*_a_ was over 30°C. There was significant variation in daily (*t* = 16.46, *df* = 90, *p* < .01) and monthly bare rock use (ANOVA: *F* = 2.43, *df* = 11, *p* = .01), but no significant variation on a seasonal basis (ANOVA: *F* = 0.57, *df* = 3, *p* = .64). Bare rock use at medium *T*_a_ occurred more frequently than those at both high and low *T*_a_.

There was no significant difference among *T*_a_ categories for the time langurs spent using branches, bare rocks, or caves (Table 3). Based on AIC, the best‐fit model selected for *T*_a_ included only branches (branch = 28.10 + 10.76 × *T*_a_, AIC = 353. 53, *df* = 88, *F* = 8.50, *p* = .004), while bare rocks and caves were both excluded from the final model, suggesting that branch use was significantly influenced by *T*_a_.

## 4 DISCUSSION

### 4.1 Activity budget and climatic factors

Most physiological processes in homeothermic animals are constrained within a narrow range of thermal conditions (Stiling, 1996). In order to keep a relatively constant body temperature, animals need to conserve body heat or acquire extra heat when cold and to dissipate heat or take heat‐avoidance measures when hot (Bicca‐Marques & Calegaro‐Marques, 1998). For animals relying on low‐energy diets, behavioral adaptations (rather than physiological) are the main thermoregulatory mechanism when facing external environmental fluctuations (DaSilva, 1992).

In the wild, primates adopt various strategies to meet their thermoregulatory needs (Anderson, 2000; Dahl & Smith, 1985; Majolo et al., 2013; Pochron, 2000; Sato, 2012). Generally, primates adopt behavioral adjustments as an adaptive strategy in response to thermal stress (Campos & Fedigan, 2009; Fan et al., 2008; Li, Ma, Zhou, & Huang, 2020; Sato, 2012, 2018; Takemoto, 2004). For example, in colder seasons, *Nomascus concolor jingdongensis* spends more time resting, which is obviously correlated to decrease its thermoregulatory costs (Fan et al., 2008). Studies on captive and free‐ranging *T. francoisi* show that resting time and total sedentary budget peak during the hottest and coldest periods, respectively (Huang et al., 2007; Kruse, 1999; Kuehler, Penny, & Bargoni, 1990; Zhou et al., 2007). This is partly thought to be related to the long digestion time required by their folivorous diet. In this study, we explore these behavioral adaptations further by examining their relationship with thermal data. Our results showed that there were significant differences in resting and total sedentary budget between *T*_a_ categories (Table 3). This implies that in addition to digestive purposes, prolonged total sedentary and resting time in langurs (Tables 2 and 3) might be related to behavioral thermoregulation. Langurs showed a preference for different supports in either high or low *T*_a_s (see Section 4.2) when sedentary. Basking and huddling behaviors both help langurs to warm up (sunbathing to absorb heat, huddling to retain it) and thus are also assumed to help with thermoregulation in other primates, such as *M. fuscata* (Ueno & Nakamichi, 2018), *T. leucocephalus* (Huang, Wei, et al., 2003), and *Rhinopithecus roxellana* (Zhang, Li, & Qi, 2011). In our study, sunbathing and huddling exclusively occurred during the coldest times of the year and showed a trend toward being more common when it is colder (Table 2), implying these behaviors had thermoregulatory properties. However, there was no significant difference in the proportions of these behaviors between *T*_a_ categories (Table 3). Therefore, the behavioral budget of *T. francoisi* might play a role in thermoregulation, which supports our first hypothesis. Although grooming was thought to indirectly influence body heat conservation by facilitating huddling formation in *M. fuscata* (Ueno & Nakamichi, 2018), our results (Table 2) showed no significant variation in grooming frequency between *T*_a_ categories (Table 3). Until new research investigates this behavior more thoroughly, we cannot conclude that this behavior is related to thermoregulation.

### 4.2 Support use and climatic factors

Thermoregulatory pressures vary seasonally; thus, animals will be subjected to significant thermal variations throughout their lifetime. Some primates select particular microhabitats as a response to thermal stress. For example, *Sapajus cay* in eastern Paraguay prefer larger trees with a wider crown, which is thought to be related to thermoregulation (Smith, Hayes, Smith, & Dickens, 2018). Additionally, sleeping sites are also selectively used by primates to avoid thermal stress (Ellison et al., 2019; Karanewsky & Wright, 2015). In our study, *T. francoisi* spent most of their time staying on branches (including rock shelter by trees and ledges) when daily *T*_a_ was highest (Table 2). Furthermore, branch use was the only variable included in the best‐fit model selected for *T*_a_, implying that langurs use branches to avoid thermal stress during the warmest parts of the day, which supports our second hypothesis.

Mature langurs are covered by black fur (Ye, Pen, Wang, & Pan, 1993). Dark pelage is associated with rapid heat gain; as a result, chimpanzees (*Pan troglodytes*) in captivity adopt a sun‐avoidance strategy by using the shade of vegetation (Duncan & Pillay, 2013). Although *T*_a_ under the dense canopy was not measured in this study, other studies have shown that shade cast by the canopy forms a microclimate. In this microclimate, wind, temperature, shade, and humidity are altered, significantly reducing heat stress (Armstrong, 1994). The shade provided by vegetation is important in habitats with temperature extremes. For example, *P. cynocephalus* are able to maintain a steady net heat balance up to 40.8°C (Funkhouser, Higgins, & Snow, 1967; Stelzner, 1988) in Amboseli National Park, Kenya. In the increasing heat stress at mid‐day, they use plots with more shade whenever possible (Stelzner, 1988). The langurs in our study most probably remained in the shade for the same thermoregulatory purpose. Prolonged branch use has also been observed in other *T. francoisi* groups in the Jinfo Mountain Reserve (Han, Wu, Zhou, Ma, & Li, 2010) and the Nonggang Nature Reserve in China (Zhou et al., 2007), as well as in close relatives in similar habitats, such as *T. delacouri* (Workman, 2010) and *T. leucocephalus* (Zhou, Huang, Tang, & Huang, 2010).

Alternative support use, including bare rocks and caves, showed no significant variation between temperature categories (Table 3). However, bare rocks and caves were frequently used during warm days and cold conditions (Table 2), similarly to observations from a previous study (Huang et al., 2004) and in a study on Delacour's langurs (Workman, 2010), suggesting they may be involved in thermoregulation, despite the lack of significance in our results. More research is required to confirm this hypothesis.

We admit that there might be limitations in our study. We report a general pattern of the behavioral adjustments and support use of François' langur based on a single group with 4 individuals, likely causing tempering of the conclusion by the small number of observed individuals. A bisexual group of François' langur includes 3–17 individuals in Vietnam (Nadler et al., 2003) and contains 4–10 individuals in Guangxi, China (Li et al., 2007). The François' langur groups outside the protected areas generally have fewer members than those within well‐protected natural reserves (Li et al., 2007). Thus, our study provides a general pattern of the behavioral ecology of François' langurs living in fragmented forest such as our study area, highlighting the need to understand the significance of behavioral adjustments and support use in behavioral thermoregulation for karst‐dwelling langurs. Moreover, it must be noted that due to the nature of the topographic conditions in this study, we were unable to physically keep a constant watch on the focal animals; the actual thermal conditions may have thus been different from what we reported. Additionally, the habitat occupied by langurs had been dramatically altered by human activity. Limestone langurs use various substrates and show a clear preference for lowland habitats with dense trees and no human presence (Li & Rogers, 2005; Workman, 2010). However, the fact that our study was unable to explain the trends in support use might be due to “active” habitat selection, as langurs may have been forced to inhabit less suitable habitats due to human disturbance. Therefore, long‐term research is required before making definitive conclusions on langurs' behavioral responses to thermal stress in limestone habitats, as primate responses to changing habitats may be site‐ and disturbance‐specific (Fimbel, 1994; Onderdonk & Chapman, 2000).

Nevertheless, the langurs' habitat investigated in this study was protected and firewood collection was prohibited, except at the foothill (Li & Rogers, 2004). Langurs therefore mostly used the hilltop and steep cliffs, and they seldom used the foothills and never used the cultivated flat land, suggesting that langurs experienced less human disturbance when they used the former habitats (Li, Ding, Huang, Jiang, & Chia, 2014). The significant correlation between prolonged resting time, total sedentary activity budget, branch use, and *T*_a_, may, therefore, have been more related to behavioral thermoregulation than habitat fragmentation and human disturbance. This study can potentially advise conservation management strategies for this specific habitat. Our results showed that langurs used beneficial supports in either high or low *T*_a_s when sedentary, suggesting that vegetation coverage is important for behavioral thermoregulation. Thus, vegetation restoration of the habitat, including gentle, earthy and moist foothill (Li & Rogers, 2005) and the flat land around the home range of the animals, is needed. It is reasonable to assume that large trees provide thermoregulatory advantages as well as food resources. Therefore, firewood collection in this habitat, including at the foothill, should be prohibited, or at least reduced, which will help improve the quality of the habitat.

## CONFLICT OF INTEREST

None declared.

## AUTHOR CONTRIBUTION

**Youbang Li:** Conceptualization (equal); Data curation (equal); Formal analysis (equal); Funding acquisition (equal); Investigation (equal); Methodology (equal); Writing‐original draft (equal). **Xiaohong Huang:** Conceptualization (equal); Methodology (equal). **Zhonghao Huang:** Conceptualization (equal); Formal analysis (equal); Funding acquisition (equal); Methodology (equal); Writing‐review & editing (equal).

## Data Availability

All data are available in the figshare repository at https://doi.org/10.6084/m9.figshare.11986890.v1

## References

[ece36249-bib-0001] Abreu, F. , De la Fuente, M. F. C. , Schiel, N. , & Souto, A. (2016). Feeding ecology and behavioral adjustments: Flexibility of a small neotropical primate *Callithrix jacchus* to survive in a semiarid environment. Mammal Research, 613, 221–229.

[ece36249-bib-0002] Altmann, J. (1974). Observational study of behavior: Sampling methods. Behavior, 49, 227–267. 10.1163/156853974x00534 4597405

[ece36249-bib-0003] Anderson, J. R. (2000). Sleep‐related behavioural adaptations in free‐ranging anthropoid primates. Sleep Medicine Reviews, 4, 355–373. 12531175 10.1053/smrv.2000.0105

[ece36249-bib-0004] Armstrong, D. V. (1994). Heat stress interaction with shade and cooling. Journal of Dairy Science, 77, 2044–2050. 7929964 10.3168/jds.S0022-0302(94)77149-6

[ece36249-bib-0005] Aujard, F. , Séguy, M. , Terrien, J. , Botalla, R. , Blanc, S. , & Perret, M. (2006). Behavioral thermoregulation in a non human primate: Effects of age and photoperiod on temperature selection. Experimental Gerontology, 41, 784–792. 16842958 10.1016/j.exger.2006.06.001

[ece36249-bib-0006] Barrett, L. , Gaynor, D. , Rendall, D. , Mitchell, D. , & Henzi, S. P. (2004). Habitual cave use and thermoregulation in chacma baboons (*Papio hamadryas ursinus*). Journal of Human Evolution, 46, 215–222. 14871563 10.1016/j.jhevol.2003.11.005

[ece36249-bib-0007] Bicca‐Marques, J. C. , & Calegaro‐Marques, C. (1998). Behavioral thermoregulation in a sexually and developmentally dichromatic neotropical primate, the black‐and‐gold howling monkey (*Alouatta caraya*). American Journal of Physical Anthropology, 106, 533–546. 9712481 10.1002/(SICI)1096-8644(199808)106:4<533::AID-AJPA8>3.0.CO;2-J

[ece36249-bib-0008] Blackshaw, J. , & Blackshaw, A. W. (1994). Heat stress in cattle and the effect of shade on production and behaviour: A review. Australian Journal of Experimental Agriculture, 34, 285–295.

[ece36249-bib-0009] Campos, F. A. , & Fedigan, L. M. (2009). Behavior adaptations to heat stress and water scarcity in white‐faced capuchins (*Cebus capucinus*) in Santa Rosa National Park, Costa Rica. American Journal of Physical Anthropology, 138, 101–111. 18711741 10.1002/ajpa.20908

[ece36249-bib-0010] Clutton‐Brock, T. H. (1975). Feeding behavior of red colobus and black and white colobus in East Africa. Folia Primatologica, 23, 165–207. 10.1159/000155671 805763

[ece36249-bib-0011] Dahl, J. F. , & Smith, E. O. (1985). Assessing variation in the social behavior of stumptail macaques using thermal criteria. American Journal of Physical Anthropology, 68, 467–477. 4083338 10.1002/ajpa.1330680403

[ece36249-bib-0012] DaSilva, G. L. (1992). The western black‐and‐white colobus as a low energy strategist: Activity budgets, energy expenditure and energy intake. Journal of Animal Ecology, 61, 79–91.

[ece36249-bib-0013] Dunbar, R. I. M. (1992). Time: A hidden constraint on the behavioural ecology of baboons. Behavioral Ecology and Sociobiology, 31, 35–49.

[ece36249-bib-0014] Dunbar, R. I. M. , Korstjens, A. H. , & Lehmann, J. (2009). Time as an ecological constraint. Biology Reviews, 84, 413–429. 10.1111/j.1469-185X.2009.00080.x 19485986

[ece36249-bib-0015] Duncan, L. M. , & Pillay, N. (2013). Shade as a thermoregulatory resource for captive chimpanzees. Journal of Thermal Biology, 38, 169–177.

[ece36249-bib-0016] Ellison, G. , Wolfenden, A. , Kahana, L. , Kisingo, A. , Jamieson, J. , Jones, M. , & Bettridge, C. M. (2019). Sleeping site selection in the nocturnal northern lesser galago (*Galago senegalensis*) supports antipredator and thermoregulatory hypotheses. International Journal of Primatology, 40, 276–296.

[ece36249-bib-0017] Fan, P. F. , Ni, Q. Y. , Sun, G. Z. , Huang, B. , & Jiang, X. L. (2008). Seasonal variations in the activity budget of *Nomascus concolor jingdongensis* at Mt. Wuliang, Central Yunnan, China: Effects of diet and temperature. International Journal of Primatology, 29, 1047–1057.

[ece36249-bib-0018] Fimbel, C. (1994). Ecological correlates of species success in modified habitats may be disturbance‐ and site‐specific: The primates of Tiwai Island. Conservation Biology, 8, 106–113.

[ece36249-bib-0019] Fuente, M. F. C. D. L. , Souto, A. , Sampaio, M. B. , & Schiel, N. (2014). Behavioral adjustments by a small neotropical primate *Callithrix jacchus* in a semiarid caatinga environment. The Scientific World Journal, 2014, 326524. 25431785 10.1155/2014/326524 PMC4241275

[ece36249-bib-0020] Funkhouser, G. E. A. , Higgins, T. , & Snow, C. C. (1967). The response of savannah baboon (*Papio cynocephalus*) to thermal stress. Life Science of Oxford, 6, 1615–1620. 10.1016/0024-3205(67)90171-3 4963372

[ece36249-bib-0021] Fusui County Annals Committee (1989). Fusui county annals. Nanning, China: Guangxi People's Publishing House.

[ece36249-bib-0022] Gestich, C. C. , Caselli, C. B. , & Setz, E. Z. F. (2014). Behavioural thermoregulation in a small neotropical primate. Ethology, 120, 331–339.

[ece36249-bib-0023] Gordon, C. J. , Fehlner, K. S. , & Long, M. D. (1986). Relationship between autonomic and behavioral thermoregulation in the golden hamster. American Journal of Physiology, 251, 320–324. 10.1152/ajpregu.1986.251.2.R320 3740314

[ece36249-bib-0024] Hafez, E. S. E. (1964). Behavioral thermoregulation in mammals and birds. International Journal of Biometeror, 7, 231–240.

[ece36249-bib-0025] Han, Z. X. , Wu, S. B. , Zhou, X. R. , Ma, J. L. , & Li, H. Q. (2010). Behaviour strategy of François' langur (*Trachypithecus francoisi*) responding factors of sunshine and rainfall in Jinfo Mountain. Journal of Anhui Agriculture Science, 38, 2968–2970.

[ece36249-bib-0026] Hendershott, R. , Rawson, R. , & Behie, A. (2018). Home range size and habitat use by Cat Ba Langurs (*Trachypithecus poliocephalus*) in a disturbed and fragmented habitat. International Journal of Primatology, 39, 547–566.

[ece36249-bib-0027] Hill, R. A. (2006). Day length seasonality and the thermal environment In Brockman D. K., & Van Schaik C. P. (Eds.), Seasonality in primates (pp. 197–214). Cambridge, UK: Cambridge University Press.

[ece36249-bib-0028] Hill, R. A. , Weingrill, T. , & Barrett, L. (2004). Indices of environmental temperature for primates in open habitats. Primates, 45, 7–13. 14608507 10.1007/s10329-003-0054-8

[ece36249-bib-0029] Huang, C. M. , Li, Y. B. , Zhou, Q. H. , & Wei, F. W. (2003). Activity patterns and their occurrences in day range of François' langur (*Trachypithecus francoisi*) in Fusui Reserve, Guangxi. Journal of Guangxi Normal University, 21, 78–82.

[ece36249-bib-0030] Huang, C. M. , Li, Y. B. , Zhou, Q. H. , & Wei, F. W. (2004). The preliminary study on behavior of cave‐leaving and entering and selection of sleeping cave of François' langur in China In Nadler T., Streicher U., & Long H. T. (Eds.), Conservation of primates in Vietnam (pp. 137–143). Hanoi, Vietnam: Haki Publishing.

[ece36249-bib-0031] Huang, C., Wei, F. , Li, M. , Li, Y., & Sun, R. ( 2003). Sleeping cave selection, activity pattern and time budget of white‐headed langurs. International Journal of Primatology, 24, 813–824.

[ece36249-bib-0032] Huang, C. M. , Wei, X. S. , Zhou, Q. H. , Li, Y. B. , & Huang, Z. H. (2007). Effects of habitat quality on activity budgets of the François' langur (*Trachypithecus francoisi*). Acta Theriologica Sinica, 27, 338–343.

[ece36249-bib-0033] Huang, C., Wu, H. , Zhou, Q., Li, Y. , & Cai, X . (2008). Feeding strategy of François' langur and white‐headed langur at Fusui, China. American Journal of Primatology, 70, 320–326. 17924424 10.1002/ajp.20490

[ece36249-bib-0034] IUCN (2008). The IUCN red list of threatened species. Retrieved from http://www.iucnredlist.org/

[ece36249-bib-0035] Karanewsky, C. J. , & Wright, P. C. (2015). A preliminary investigation of sleeping site selection and sharing by the brown mouse lemur *Microcebus rufus* during the dry season. Journal of Mammalogy, 96, 1344–1351.

[ece36249-bib-0036] Kobbe, S. , Nowack, J. , & Dausmann, K. H. (2014). Torpor is not the only option: Seasonal variations of the thermoneutral zone in a small primate. Journal of Comparative Physiology B‐Biochemical Systemic and Environmental Physiology, 1846, 789–797. 10.1007/s00360-014-0834-z 24942312

[ece36249-bib-0037] Kruse, D. H. (1999). Aspects of the behavior of François' langurs (*Trachypithecus francoisi*) in captivity. Ph. D. dissertation. Portland, OR: Portland State University.

[ece36249-bib-0038] Kuehler, C. , Penny, C. , & Bargoni, R. (1990). Zoological Society of San Diego François Langur (*Presbytis francoisi francoisi*) Master Plan. San Diego, CA: Zoological Society of San Diego.

[ece36249-bib-0039] Li, Y., Ding, P. , Huang, C. , Jiang, P. , & Chia, E. (2014). Ranging patterns of François' langur in a fragmented habitat in Fusui Reserve, Guangxi Province, China. Mammalia, 78, 75–84.

[ece36249-bib-0040] Li, Y., Huang, C., Ding, P. , Tang, Z. , & Wood, C. (2007). Dramatic decline of François's langur (*Trachypithecus francoisi*) in Guangxi Province, China. Oryx, 41, 38–43.

[ece36249-bib-0041] Li, Y., Ma, G., Zhou, Q. , & Huang, Z. (2020). Ranging patterns and foraging patch utilization of Assamense macaques inhabiting limestone forests in southwest Guangxi, China. Global Ecology and Conservation, 21, e00816.

[ece36249-bib-0042] Li, Z. Y. , & Rogers, M. E. (2004). Habitat quality and activity budgets of white‐headed langurs in Fusui. China. International Journal of Primatology, 25(1), 41–54. 10.1159/000086020 16088186

[ece36249-bib-0043] Li, Z., & Rogers, M. E. (2005). Are limestone hills a refuge or essential habitat for white‐headed langurs in Fusui, China? International Journal of Primatology, 26, 437–452. 10.1159/000086020 16088186

[ece36249-bib-0044] Liu, Z. , Groves, C. , Yuan, D. , & Meiman, J. (2004). South China karst aquifer storm‐scale hydrochemistry. Ground Water, 42, 491–499. 15318771 10.1111/j.1745-6584.2004.tb02617.x

[ece36249-bib-0045] Ma, G. , Bai, C. M. , Wang, X. J. , Majeed, M. Z. , & Ma, C. S. (2018). Behavioral thermoregulation alters microhabitat utilization and demographic rates in ectothermic invertebrates. Animal Behavior, 142, 49–57.

[ece36249-bib-0046] Majolo, B. , McFarland, R. , Young, C. , & Qarro, M. (2013). The effect of climatic factors on the activity budgets of barbary macaques (*Macaca sylvanus*). International Journal of Primatology, 34, 500–514.

[ece36249-bib-0047] Martin, P. , & Bateson, P. (1986). Measuring behavior: An introductory guide (2nd ed.). Cambridge, UK: Cambridge University Press.

[ece36249-bib-0048] McFarland, R. , Fuller, A. , Hetem, R. S. , Mitchell, D. , Maloney, S. K. , Henzi, S. P. , & Barrett, L. (2015). Social integration confers thermal benefits in a gregarious primate. Journal of Animal Ecology, 84, 871–878. 25581128 10.1111/1365-2656.12329

[ece36249-bib-0049] McLester, E. , Brown, M. , Stewart, F. , & Piel, A. K. (2019). Food abundance and weather influence habitat‐specific ranging patterns in forest‐ and savanna mosaic‐dwelling red‐tailed monkeys (*Cercopithecus ascanius)* . American Journal of Physical Anthropology, 170, 217–231. 31423563 10.1002/ajpa.23920

[ece36249-bib-0050] Mount, L. E. (1979). Adaptation to thermal environment. London, UK: Arnold.

[ece36249-bib-0051] Nadler, T. , Momberg, F. , Dang, N. X. , & Lormee, N. (2003). Vietnam primate conservation status review 2002: Part 2: leaf monkeys. Hanoi, Vietnam: Fauna & Flora International‐Vietnam Program and Frankfurt Zoological society.

[ece36249-bib-0052] Ogawa, H. , & Wada, K. (2011). Shape of, and body direction in, huddles of Japanese macaques *Macaca fuscata* in Arashiyama, Japan. Primates, 52, 229–235. 21442467 10.1007/s10329-011-0248-4

[ece36249-bib-0053] Onderdonk, D. A. , & Chapman, C. A. (2000). Coping with forest fragmentation: The primates of Kibale National Park, Uganda. International Journal of Primatology, 21, 587–611.

[ece36249-bib-0054] Pochron, S. T. (2000). Sun avoidance in the yellow baboons (*Papio cynocephalus cynocephalus*) of Ruaha National Park, Tanzania. Variations with season, behavior and weather. International Journal of Biometeorology, 44, 141–147. 11049003 10.1007/s004840000058

[ece36249-bib-0055] Post, D. G. (1981). Activity patterns of yellow baboons (*Papio cynocephalus*) in the Amboseli National Park, Kenya. Animal Behavior, 29, 357–374.

[ece36249-bib-0056] Pruetz, J. D. (2007). Evidence of cave use by savanna chimpanzees (*Pan troglodytes verus*) at Fongoli, Senegal: Implications for thermoregulatory behavior. Primates, 48, 316–319. 17624495 10.1007/s10329-007-0038-1

[ece36249-bib-0057] Sade, D. S. (1972). A longitudinal study of social behavior of rhesus monkeys In Tuttle R., & Atherton A. (Eds.), The function and evolutionary biology of primates. New York, NY: University of Chicago Press.

[ece36249-bib-0058] Sato, H. (2012). Diurnal resting in brown lemurs in a dry deciduous forest, northwestern Madagascar: Implications for seasonal thermoregulation. Primates, 53, 255–263. 22388421 10.1007/s10329-012-0301-y

[ece36249-bib-0059] Sato, H. (2018). Predictions of seed shadows generated by common brown lemurs (*Eulemur fulvus*) and their relationship to seasonal behavioral strategies. International Journal of Primatology, 39, 377–396.

[ece36249-bib-0060] Smith, A. U. (1958). Life at low temperature. Nature, 182, 911–913.

[ece36249-bib-0061] Smith, R. L. , Hayes, S. E. , Smith, P. , & Dickens, J. K. (2018). Sleeping site preferences in *Sapajus cay* Illiger 1815 (Primates: Cebidae) in a disturbed fragment of the Upper Paraná Atlantic Forest, Rancho Laguna Blanca, Eastern Paraguay. Primates, 59, 79–88. 28825150 10.1007/s10329-017-0626-7

[ece36249-bib-0062] Stanford, C. B. (1991). The capped langur in Bangladesh: Behavior ecology and reproductive tactics. New York, NY: Karger.

[ece36249-bib-0063] Stelzner, J. K. (1988). Thermal effects on movement patterns of yellow baboons. Primates, 29, 91–105.

[ece36249-bib-0064] Stelzner, J. K. , & Hausfater, G. (1986). Posture, microclimate, and thermoregulation in yellow baboons. Primates, 27, 449–463.

[ece36249-bib-0065] Stewart, F. A. , Piel, A. K. , Azkarate, J. C. , & Pruetz, J. D. (2018). Savanna chimpanzees adjust sleeping nest architecture in response to local weather conditions. American Journal of Physical Anthropology, 166, 549–562. 29989162 10.1002/ajpa.23461

[ece36249-bib-0066] Stiling, P. D. (1996). Ecology: Theories and applications (2nd ed.). Upper Saddle River, NJ: Prentic Hall.

[ece36249-bib-0067] Struhsaker, T. (1975). The red colobus monkey. Chicago, IL: Chicago University Press.

[ece36249-bib-0068] Takemoto, H. (2004). Seasonal change in terrestriality of chimpanzees in relation to microclimate in the tropical forest. American Journal of Physical Anthropology, 124, 81–92. 15085550 10.1002/ajpa.10342

[ece36249-bib-0069] Thompson, C. L. , Williams, S. H. , Glander, K. E. , & Vinyard, C. J. (2016). Measuring microhabitat temperature in arboreal primates: A comparison of on‐animal and stationary approaches. International Journal of Primatology, 37, 495–517.

[ece36249-bib-0070] Tian, B. P. , & Zhang, L. Y. (1993). Prevention and treatment of disease In Ye Z. Z. (Ed.), Biology of leaf monkey (Presbytis) (pp. 571–604). Kunming, China: Yunnan Science and Technology Press.

[ece36249-bib-0071] Tuff, K. T. , Tuff, T. , & Davies, K. F. (2016). A framework for integrating thermal biology into fragmentation research. Ecology Letter, 19, 361–374. 10.1111/ele.12579 PMC4794773 26892491

[ece36249-bib-0072] Ueno, M. , & Nakamichi, M. (2018). Grooming facilitates huddling formation in Japanese macaques. Behavioral Ecology and Sociobiology, 72, 98–107.

[ece36249-bib-0073] van Soest, P. J. (1982). Nutritional ecology of the ruminant. Ithaca, NY: Cornell University Press.

[ece36249-bib-0074] Vogt, J. L. (1978). The social behavior of a marmoset *Saguinus fuscicollis* group II: Behavior patterns and social interaction. Primates, 19, 287–300. 97193

[ece36249-bib-0075] Whiteman, H. H. , & Buschhaus, N. L. (2003). Behavior thermoregulation in field populations of amphibian larvae In Ploger B. J., & Yasukawa K. (Eds.), Exploring animal behavior in laboratory and field‐ an hypothesis‐testing approach to development, causation, function, and evolution of animal behavior (pp. 79–84). London, UK: Academic Press.

[ece36249-bib-0076] Workman, C. (2010). The foraging ecology of the Delacour's langur (*Trachypithecus delacouri*) in Van Long Nature Reserve, Vietnam. Ph.D. Desertation. Duke University. 10.1002/ajp.20785 20027639

[ece36249-bib-0077] Xue, Y. G. (2000). Guangxi tropical flora. Guilin, China: Guangxi Normal University Press.

[ece36249-bib-0078] Ye, Z. Z. , Pen, Y. Z. , Wang, H. , & Pan, R. L. (1993). Anatomy In Ye Z. Z. (Ed.), Biology of leaf monkey (*Presbytis*) (pp. 571–604). Kunming, China: Yunnan Science and Technology Press.

[ece36249-bib-0079] Zhang, P. , Li, B. G. , & Qi, X. G. (2011). Sleeping cluster patterns and retiring behaviors during winter in a free‐ranging band of the Sichuan snub‐nosed monkey. Primates, 52, 221–228. 21350935 10.1007/s10329-011-0241-y

[ece36249-bib-0080] Zhou, Q. H. , Huang, C. M. , & Fan, Y. (2001). Time budgets of *Presbytis francoisi* . Journal of Guangxi Normal University, 19, 79–82.

[ece36249-bib-0081] Zhou, Q. H. , Huang, H. L. , Tang, X. P. , & Huang, C. M. (2010). Seasonal variations in the activity budgets of the white‐headed langur. Acta Theriologica Sinica, 30, 449–455.

[ece36249-bib-0082] Zhou, Q., Huang, Z. , Wei, X. , Wei, F. , & Huang, C . (2009). Factors influencing interannual and intersite variability in the diet of *Trachypithecus francoisi* . International Journal of Primatology, 30, 583–599.

[ece36249-bib-0083] Zhou, Q., Luo, B. , Wei, F. , & Huang, C. (2013). Habitat use and locomotion of the François' langur (*Trachypithecus francoisi*) in limestone habitats of Nonggang, China. Integrative Zoology, 8, 346–355. 24344958 10.1111/j.1749-4877.2012.00299.x

[ece36249-bib-0084] Zhou, Q., Wei, F. , Huang, C. , Li, M. , Ren, B. , & Luo, B. (2007). Seasonal variation in the activity patterns and time budgets of *Trachypithecus francoisi* in the Nonggang Nature Reserve, China. International Journal of Primatology, 28, 657–671.

[ece36249-bib-0085] Zuur, A. F. , Leno, E. N. , & Elphick, C. S. (2010). A protocol for data exploration to avoid common statistical problems. Methods in Ecology and Evolution, 1, 3–14.

